# Cdk2 phosphorylation of Bcl-xL after stress converts it to a pro-apoptotic protein mimicking Bax/Bak

**DOI:** 10.1038/cddiscovery.2015.66

**Published:** 2016-01-18

**Authors:** J Megyesi, A Tarcsafalvi, NSHL Seng, R Hodeify, PM Price

**Affiliations:** 1 Department of Internal Medicine, Division of Nephrology, University of Arkansas for Medical Sciences, Little Rock, AR, USA; 2 Department of Physiology and Biophysics, University of Arkansas for Medical Sciences, Little Rock, AR, USA; 3 Central Arkansas Veterans Healthcare System, Little Rock, AR, USA; 4 Department of Physiology and Biophysics, Weill Cornell Medical College in Qatar, Doha, Qatar

## Abstract

Apoptosis is a regulated form of cell death that proceeds by defined biochemical pathways. Most apoptosis is controlled by interactions between pro-survival and pro-apoptotic Bcl-2 family proteins in which death is often the consequence of permeabilization of the mitochondrial outer membrane. Many drugs affect this equilibrium to favor apoptosis but this process is not completely understood. We show that the chemotherapeutic drug cisplatin initiates an apoptotic pathway by phosphorylation of a pro-survival Bcl-2 family member, Bcl-xL, by cyclin-dependent kinase 2. The phosphorylation occurred at a previously unreported site and its biologic significance was demonstrated by a phosphomimetic modification of Bcl-xL that was able to induce apoptosis without addition of cisplatin. The mechanism of cell death induction was similar to that initiated by pro-apoptotic Bcl-2 family proteins, that is, phosphorylated Bcl-xL translocated to the mitochondrial membrane, and formed pores in the membrane. This initiated cytochrome *c* release and caspase activation that resulted in cell death.

## Introduction

Proteins of the Bcl-2 family are important regulators of apoptotic cell death in which pro-apoptotic members, such as Bax and Bak, can initiate cell death pathways and pro-survival members, such as Bcl-xL, interact with pro-apoptotic proteins to inhibit these activities.^1^ Although these proteins are functionally different, Bax and Bcl-xL have similar sequence homology, and are expected to have the same three-dimensional conformation.^2^ In the cytoplasmic forms of these proteins, the transmembrane domain is tucked within a hydrophobic groove on the surface while on the opposite side and masked by an unstructured loop, is a minor groove. The minor groove was proposed to be a trigger site for Bax activation.^3^

Activation of Bax is initiated by shifting the unstructured loop and allosterically displacing the transmembrane region from the other side of the protein.^3–5^ Although Bax exists primarily as a monomer in the cytosol of healthy cells,^6^ active Bax is translocated to the mitochondria^7^ and after its insertion into the outer membrane it oligomerizes^8^ ultimately causing the release of mitochondrial cytochrome *c*.^9^ The first indication that oligomerization of pro-apoptotic proteins was a mechanism for mitochondrial membrane permeabilization was the observation that Bax and Bak coalesced into mitochondrial-associated foci during apoptosis,^10^ which led to the proposal of the pore hypothesis.^11^ The mechanism of pore formation is still the subject of intense research. One important question is that although the structure of the pro-survival protein Bcl-xL resembles that of pro-apoptotic proteins, Bcl-xL prevents rather than participates in pore formation. Many chemotherapeutic drugs disturb the Bcl-2 family protein equilibrium to affect cancer cell death.^12^ Cisplatin is one of the most effective chemotherapeutic drugs but its use is compromised by its nephrotoxicity.^13,14^ The mechanism of cell death induced by cisplatin is not completely understood, but its cytotoxicity in cancer cells has been attributed to its action to promote DNA damage.^15^ In normal kidney cells, but not in cancer cells, we found that cisplatin cytotoxicity could be prevented by inhibition of cyclin-dependent kinase 2 (Cdk2), a serine–threonine protein kinase associated with cell cycle progression.^16^ We demonstrated that markedly increased Cdk2 activity was associated with cisplatin-induced cell death both *in vitro* and *in vivo.* ^17^ Other studies also showed that increased Cdk2 activity was sometimes associated with apoptosis and that activated caspases could promote this increase.^18–20^

We now show that after cisplatin exposure, Cdk2 phosphorylated Bcl-xL at a previously unreported site in its unstructured loop. This initiated an apoptotic pathway in which phosphorylation converted this pro-survival protein into a protein capable of initiating apoptosis, even in the absence of cisplatin. Our data suggests that the mechanism of this cell death is similar to that initiated by the pro-apoptotic Bcl-2 family proteins, that is, phosphorylation caused a conformational change in the molecule, the protein localized primarily to the mitochondrial membrane and phospho-Bcl-xL aggregates formed pores in the membrane, initiating cytochrome *c* release and caspase activation that resulted in cell death. This apoptotic pathway demonstrates a unique mechanism linking cell cycle to cell death and also provides insight into mitochondrial pore formation by Bcl-2 family proteins.

## Results

### Cdk2 activity is required for an apoptotic pathway

**Figure 1:**
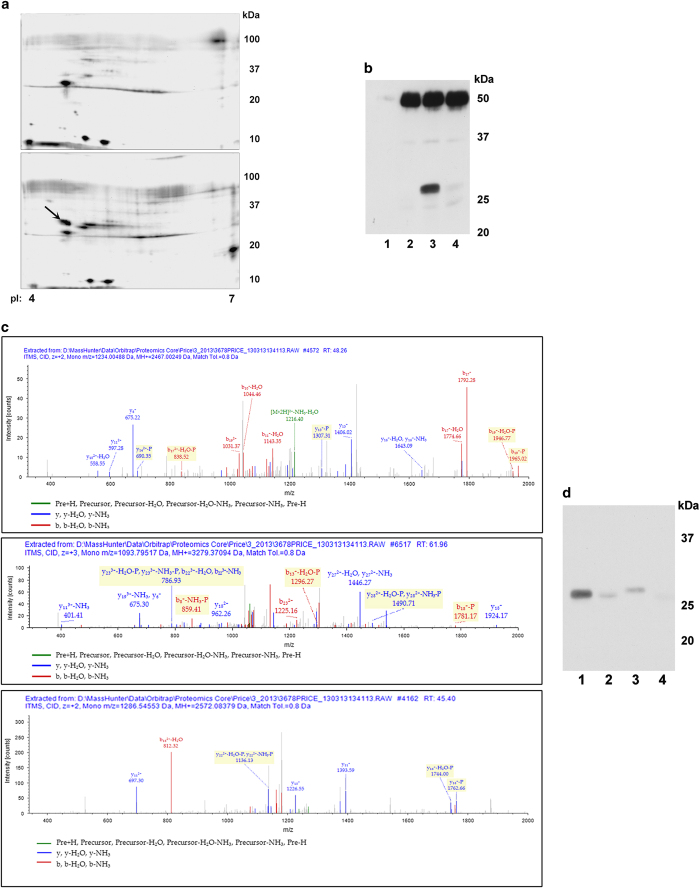
(**a**) Two-dimensional gel electrophoresis. Substrates from TKPTS cells were kinased by as-Cdk2 isolated either from untreated (top panel) or 20 h cisplatin-treated (lower panel) TKPTS cells transduced 24 h previously with as-Cdk2 and cyclinA expression adenoviruses. Proteins were separated by pI (pH 4–7, left to right) and size (100–10 kDa, top to bottom). Protein later identified as Bcl-xL labeled with arrow. (**b**) Thiophosphophorylation of His-tagged ΔC human Bcl-xL by Cdk2. wild-type or as-Cdk2 was used to kinase Bcl-xL using *N*^*6*^-benzyladenosine-5ʹ-O-(3-thiotriphosphate). Thiophosphorylation was identified in proteins treated with *p*-nitrobenzyl mesylate (PNBM) using *α*-thiophosphoester antibody. Lane 1, control immunoprecipitation performed without antibody; lane 2, wild-type Cdk2; lane 3, as-Cdk2; lane 4, as-Cdk2 with added purvalanol Cdk inhibitor. (**c**) Thiophosphorylated residues identified in Bcl-xL after kinase reaction with as-Cdk2 and *N*^*6*^-benzyladenosine-5ʹ-O-(3-thiotriphosphate). Residues identified with SEQUEST (v.1.20; a registered trademark of the University of Washington), fragment match tolerance used for search: 0.8  Da, fragments used for search: b; b-H_2_O; b-NH_3_; y; y-H_2_O; y-NH_3_. Bcl-xL protein coverage 91.42%. All peptides containing thiophosphorylated residues are shown. Modified residues are shown in red. Peptide 19 (panel 1) contained thiophosphorylated serine-18, corresponding to S72 of Bcl-xL; Peptide 25 (panel 2) contained thiophosphorylated serine-19, corresponding to S73 of Bcl-xL; Peptide 34 (panel 3) contained thiophosphorylated serine-15, corresponding to S74 of Bcl-xL. (**d**) Thiophosphorylation of wild-type and serine-mutated Bcl-xLs. As-Cdk2 was used to kinase wild-type Bcl-xL (lanes 1, 2) and mutated Bcl-xL (serine 73 to aspartic acid (S73D), lane 3; serines 72, 73, 74 to alanines (S72,73,74A), lane 4). Thiophosphorylation was identified in proteins treated with PNBM using *α*-thiophosphoester antibody. Purvalanol was added to one reaction (lane 2).

We previously showed that both cisplatin- and ER stress-initiated apoptotic pathways *in vitro* and *in vivo* required Cdk2 activity.^17,21^ To determine potential substrates of Cdk2 that could affect cell death pathways, analog-sensitive Cdk2 (as-Cdk2) was isolated from untreated cells and cells exposed to cisplatin. Proteins from post-nuclear supernatants were kinased by as-Cdk2 and *N* ^6^-benzyl-[*γ*-^32^P]ATP and separated by two-dimensional electrophoresis. The resultant autoradiograms showed a radiolabeled protein present only in the reaction using as-Cdk2/cyclinA from cisplatin-exposed cells with a pI of ~4.5 and molecular weight of ~30 kDa (Figure 1a). The excised proteins contained Bcl-xL with a calculated molecular weight of 26.0 kDa and pI of 4.93. This protein is the most abundant pro-survival Bcl-2 family member present in kidney or cultured kidney proximal tubule cells (Supplementary Figure S1). Other proteins in the analysis had either molecular weights or pIs that did not coincide with the approximate values coinciding with the electrophoretic radiolabeled spot. We confirmed that as-Cdk2 could phosphorylate Bcl-xL using His-tagged human Bcl-xL and *N* ^*6*^-benzyladenosine-5ʹ-O-(3-thiotriphosphate), in which thiophorylation was detected by western blots (Figure 1b). Without immunoprecipitated as-Cdk2 (lane 1) or with immunoprecipitated wild-type Cdk2 (lane 2), there was no thiophosphate labeling. Thiophosphorylation of Bcl-xL was apparent using as-Cdk2 (lane 3, band at ~30 kDa), which was inhibited when purvalanol, a Cdk inhibitor, was included in the reaction (lane 4). The band at ~50 kDa is immunoglobulin heavy chain.

Thiophosphorylated residues were identified in Bcl-xL by mass spectrometry in which 91.42% of the protein was sequenced. Three peptides were identified containing thiophosphorylated residues (Figure 1c). These residues were serines located at positions 72 (panel 1), 73 (panel 2) and 74 (panel 3) of full-length Bcl-xL. In addition, post-translational modifications were detected at position 69 (phosphothreonine, panel 1), position 62 (phosphoserine, panel 2) and position 73 (phosphoserine, panel 3). Since these residues were not thiophosphorylated, it is likely they arose from phosphorylation with a bacterial kinase since the Bcl-xL substrate was purified from bacterial sources. To confirm that the residues at 72–74 represented those kinased by as-Cdk2, the serine at position 73 was mutated to aspartic acid and serines at positions 72, 73 and 74 were mutated to alanine. As shown (Figure 1d), compared with the amount of thiophosphorylation using non-mutated Bcl-xL (lane 1), the S73D mutation (lane 3) severely reduced thiophosphorylation and the S72,73,74A mutations (lane 4) eliminated thiophosphorylation. The addition of the Cdk inhibitor purvalanol in the reaction (lane 2) also severely reduced thiophosphorylation. It can also be noted that the S73D mutation caused a marked change in the mobility of the protein, although the molecular weight was only slightly increased (*cf*., lanes 1 and 2 with lane 3).

### Phosphomimetic Bcl-xL is cytotoxic

**Figure 2:**
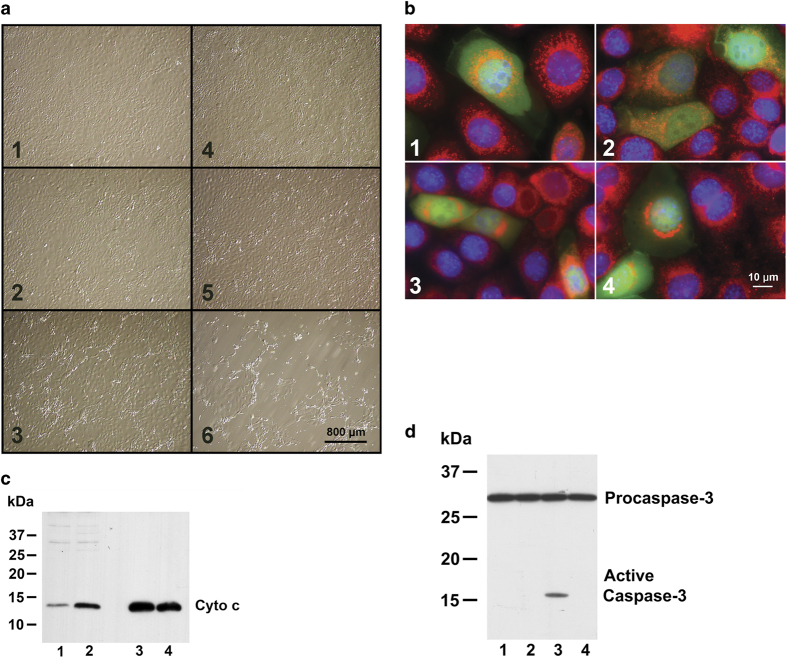
(**a**) Morphology of cultured TKPTS cells. Cells were grown to ~50% confluency, infected with adenoviruses and photographed either 48 h (panels 1–3) or 72 h (panels 4–6) later. Cells were either uninfected (panels 1, 4), infected with wild-type Bcl-xL expression adenovirus (panels 2, 5), or with S73D adenovirus (panels 3, 6). Photographs taken with ×10 objective. (**b**) Morphologic changes in mitochondria after S73D Bcl-xL expression. Cells were infected with adenoviruses that were co-expression vectors for both GFP and Bcl-xL (either wild-type, panels 1,2, or S73D, panels 3, 4). Cells positive for GFP co-expressed Bcl-xL and their mitochondrial morphology can be compared with non-expressing cells (GFP negative) in the same photomicrograph. (**c**) Cytochrome *c* in mitochondria and cytoplasm. Immunoblot for cytochrome *c* content in the cytoplasmic (lanes 1, 2) and mitochondrial (lanes 3, 4) fractions 3 days after transduction with adenovirus expressing wild-type (lanes 1, 3) and S73D Bcl-xL (lanes 2, 4). For the cytoplasmic fraction, 100 *μ*g protein was loaded, and for the mitochondrial fraction 10 *μ*g protein was loaded. (**d**) Caspase-3 activation after Bcl-xL expression. Three days after adenoviral transduction, TKPTS cells were collected and the post-nuclear supernatant analyzed by western blot for pro-caspase-3 and activated caspase-3. Proteins were isolated from untreated control (lane 1), wild-type Bcl-xL adenovirus transduced (lane 2) and S73D Bcl-xL adenovirus transduced (lanes 3, 4) cultures. In addition, some cultures were also treated with 5 *μ*M zVAD, a pan-caspase inhibitor (lane 4).

The effect of phosphomimetic Bcl-xL (S73D) compared with wild-type was determined using adenovirus expression vectors in mouse kidney proximal tubule epithelial cells (TKPTS). The S73D Bcl-xL induced changes characteristic of apoptosis and/or cell death in cultured cells even without the addition of cisplatin. Morphologically, many cells transduced with S73D Bcl-xL adenovirus showed signs of cytotoxicity starting at 48 h after virus addition, which progressed with time (Figure 2a). Uninfected cells (panels 1, 4) and cells expressing with wild-type Bcl-xL (panels 2, 5) had similar morphologies 48 and 72 h after transduction. After S73D Bcl-xL transduction (panels 3, 6), many cells showed signs of apoptosis, such as shrinking and blebbing, and detachment from the cell monolayer.

Expression of S73D Bcl-xL also affected mitochondrial morphology (Figure 2b). Expression of wild-type Bcl-xL (panels 1, 2) did not affect mitochondrial morphology or localization (*cf*., Mitotracker Red-stained mitochondria in panels 1 and 2 with cells co-expressing Bcl-xL and GFP in panels 1 and 2). However, expression of S73D Bcl-xL (panels 3, 4) caused perinuclear clustering of the mitochondria, a morphologic change reported to be associated with the first stages of apoptosis, prior to the release of cytochrome *c*.^22–24^

Three days after transduction of TKPTS cells with wild-type Bcl-xL expression adenovirus (Figure 2c, lanes 1, 3) or S73D Bcl-xL adenovirus (Figure 2c, lanes 2, 4), cytochrome *c* was present in both mitochondrial fractions (lanes 3, 4), which was released into the cytoplasm (lanes 1, 2) preferentially from the S73D Bcl-xL-expressing cells (lane 2). The same samples were processed using western blots for cytoplasmic and mitochondrial marker proteins (Supplementary Figures S2A and B).

Downstream effects of cytochrome *c* release from mitochondria include caspase activation, and caspase-3 activation is one of the terminal steps in this cascade (Figure 2d). There was no activation of caspase-3 either in control cells (lane 1) or in cells expressing wild-type Bcl-xL (lane 2), but it was activated in cells expressing S73D Bcl-xL (lane 3). The inclusion of zVAD-fmk, a pan-caspase inhibitor, in one culture expressing S73D Bcl-xL prevented caspase-3 activation (lane 4). Similarly, caspase activation was assessed by binding of Red-VAD after Bcl-xL transduction (Supplementary Figure S3A) in which binding of Red-VAD in cells transduced with wild-type or phosphorylation-defective Bcl-xL was similar to that in control cells, but binding in S73D Bcl-xL-expressing cells was similar to the binding in cisplatin-treated cells.

Cells were analyzed for cell cycle parameters by FACS (Supplementary Figure S3B) in which the Sub-G_0_/G_1_ fraction was defined as the fraction of apoptotic cells.^25^ The results confirmed that the percentage of apoptotic cells in wild-type or phosphorylation-defective Bcl-xL was similar to that in untreated cells, whereas apoptosis was induced by cisplatin exposure or expression of S73D Bcl-xL. This analysis also confirmed that zVAD-fmk prevented induction of apoptosis by S73D Bcl-xL.

### Phosphorylation-defective Bcl-xL protects from cisplatin cytotoxicity

**Figure 3:**
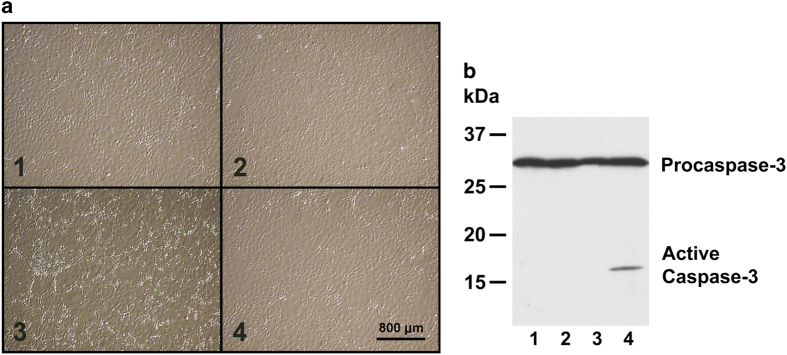
(**a**) Morphology of cells after cisplatin exposure. Representative areas of cultured cells photographed with Hoffman modulation optics. Cells were grown to ~50% confluency and either were not transduced with adenoviruses (panels 1, 3) or transduced with S72,73,74A Bcl-xL expression adenovirus (panels 2, 4). After 24 h, some cultures were exposed to 25 *μ*M cisplatin for an additional 24 h (panels 3, 4). All cells were photographed at the same time after splitting. Photographs taken with ×10 objective. (**b**) Caspase-3 activation after Bcl-xL expression and cisplatin exposure. Three days after adenoviral transduction, TKPTS cells were collected and the post-nuclear supernatant was analyzed by western blot for pro-caspase-3 and activated caspase-3. Proteins were isolated from untreated controls (lane 1), cultures exposed to 25 *μ*M cisplatin (lanes 3, 4) and transduced with S72,73,74A Bcl-xL expression adenovirus (lanes 2, 3).

Preventing phosphorylation of Bcl-xL by substituting serines 72, 73 and 74 with alanine protected from cisplatin cytotoxicity, including changes in morphology and activation of caspase-3. By analysis of morphology (Figure 3a), control cells (panel 1) and cells expressing S72,73,74A Bcl-xL (panel 2) had similar morphology in which most cells were flat and contained within the monolayer. Cells exposed to cisplatin (panel 3) had a cytotoxic morphology, and many cells were detached from the monolayer. In contrast to panel 3, cells exposed to cisplatin and expressing S72,73,74A Bcl-xL (panel 4) had a morphology similar to control. Activation of caspase-3 (Figure 3b) was not detected in control cells (lane 1) or in cells expressing S72,73,74A Bcl-xL (lane 2). Cells exposed to cisplatin had caspase-3 activation (lane 4), which was prevented by expression of S72,73,74A Bcl-xL with the cisplatin (lane 3). The phosphorylation-defective Bcl-xL also protected from cisplatin apoptosis using the criteria of cell cycle FACS analysis (Supplementary Figure S3B).

### Phosphomimetic Bcl-xL results in high molecular multimers on the mitochondrial membrane

**Figure 4:**
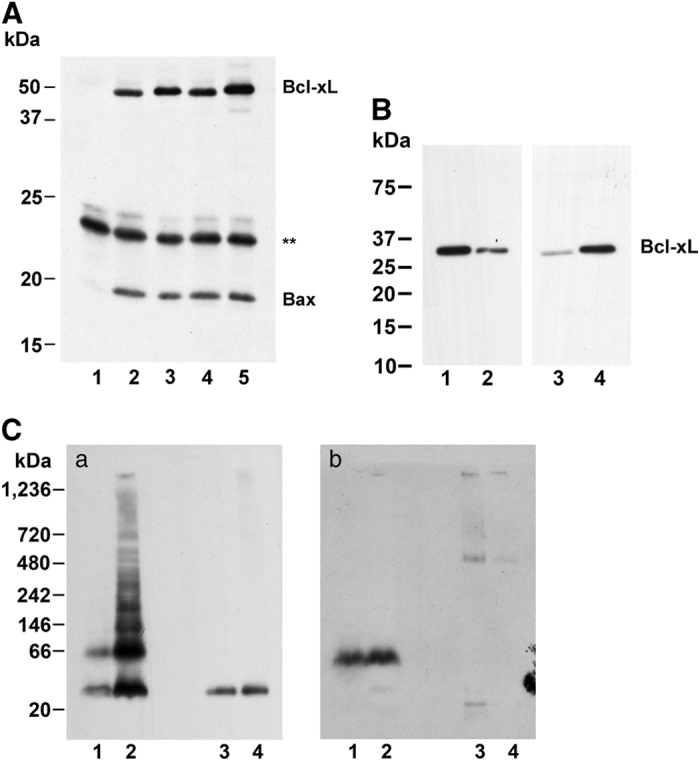
(**A**) Bax and Bcl-xL interaction. Binding of wild-type and S73D Bcl-xL to immobilized Bax. Bax was immobilized to anti-Bax antibody bound to Protein-G-Dynabeads and incubated with either wild-type (lanes 2, 4) or S73D (lanes 1, 3, 5) Bcl-xL. One incubation was performed without anti-Bax antibody (lane 1). Bax was obtained either from untreated cells (lanes 1–3) or from cells treated with tBid-expression adenovirus (lanes 4, 5). Bound proteins were eluted, electrophoresed by PAGE and probed for Bcl-xL. After localization of Bcl-xL, the membranes were probed for Bax. Asterisk, non-specific band from Protein-G-Dynabeads. (**B**) Bcl-xL on mitochondria and in cytoplasm. Bcl-xL immunoblot showing relative distribution of Bcl-xL in mitochondrial and cytoplasm fractions. Legend is the same as Figure 2c. For the cytoplasmic fraction, 10 *μ*g protein was loaded, and for the mitochondrial fraction 1 *μ*g protein was loaded. (**C**) Bcl-xL and Bax on mitochondria. Mitochondria were isolated from TKPTS cells and proteins extracted with 1% *n*-dodecyl-*β*-*D*-maltoside. Proteins from mitochondria (lanes 1, 2) and cytoplasm (lanes 3, 4) were electrophoresed on BN-PAGE. Cells were transduced with either wild-type (lanes 1, 3) or S73D (lanes 2, 4) Bcl-xL adenovirus and incubated 2 days after transduction before harvesting. Western blots were probed for Bcl-xL (**a**) or Bax (**b**).

Interaction of Bax with wild-type and S73D Bcl-xL was performed using Bax immobilized on anti-Bax Dynabeads and assay of the bound proteins by western analysis (Figure 4a). Without addition of anti-Bax antibody to the Protein-G-Dynabeads (lane 1), neither Bcl-xL nor Bax was bound. Using either wild-type Bcl-xL (lanes 2, 4) or S73D Bcl-xL (lanes 3, 5) with either inactive Bax (lanes 2, 3) or tBid-activated Bax (lanes 4, 5) resulted in identical binding of Bcl-xL. The cytoplasmic and mitochondrial fractions (Figure 2c) were also probed for Bcl-xL (Figure 4b). While wild-type Bcl-xL was primarily localized in the cytoplasm (*cf.*, lanes 1, 3), S73D Bcl-xL was primarily localized on mitochondria (*cf.,* lanes 2, 4).

Last, we examined whether either Bcl-xL or Bax could form oligomers that were associated with the mitochondrial membrane (Figure 4c), which could indicate their assembly into pores. The cells were transduced with either wild-type (lanes 1, 3) or S73D (lanes 2, 4) Bcl-xL adenovirus and incubated 2 days after transduction before harvesting. Proteins were separated into mitochondrial (lanes 1, 2) and cytoplasmic (lanes 3, 4) fractions and resolved with non-denaturing BN-PAGE. Immunoblots were probed for Bcl-xL (Figure 4C, panel a), stripped and reprobed for Bax (Figure 4C, panel b). Wild-type Bcl-xL protein was equally distributed between mitochondria and cytoplasm, with monomers and dimers present on the mitochondria, and only monomers in the cytoplasm. S73D Bcl-xL was concentrated on the mitochondria, and was present in the cytoplasm as a monomer, but oligomers were apparent in the mitochondria fraction. Bax was primarily associated with the mitochondrial membrane (panel b, lanes 1, 2) and was not present as oligomers, although it was likely associated with the Bcl-xL present in the mitochondria fraction.

## Discussion

Cdk2 is one of a family of serine–threonine protein kinases that drive the cell cycle. We and others showed that Cdks also actively participate in apoptosis from diverse causes including cisplatin, ER stress agents, TGF*β*1, CD437, DNA damage, glucocorticoids and growth factor deprivation.^17,26–32^ Protein phosphorylation by sequential activation of different Cdks regulates progression through the cell cycle presumably by recognition of different substrates and thus the regulation of their activities; pairing of Cdk with different cyclins could account for some of the preferential substrate selection.^33^ Studies with gene targeting in mice,^34^ and in fission yeast,^35^ led to minimal models of the cell cycle in which the importance of Cdk activity thresholds and localization may supercede substrate specificity.^36,37^ The amino acid sequence in Bcl-xL that is phosphorylated by Cdk2 after cisplatin exposure (Figure 1) does not correspond to the canonical recognition sequence of Cdk,^38,39^ but evidence of minor phosphorylation of serine residues in a similar triplet sequence was reported to occur in p53 by Cdk1-cyclin B^40^ and Cdk2-cyclinA.^41^ Cdk2 phosphorylation of serines at positions 72, 73 and 74 in Bcl-xL was demonstrated by several criteria. First, potentially contaminating kinases were selected against by virtue of kination using analog-sensitive Cdk2 and thiophosphorylated ATP analog (Figure 1b). Second, mutation of the proposed sites eliminated phosphorylation (Figure 1d). Third, the phosphorylation elicited a biochemical change in the substrate that had appropriate biologic consequences. Here, a phosphomimetic Bcl-xL (S73D) caused an apoptotic cell death similar to that caused by cisplatin, without the necessity of cisplatin exposure (Figure 2).

Programmed cell death, or apoptosis, proceeds by pathways that often are dependent on mitochondrial outer membrane (MOM) permeabilization,^42^ in which pro-apoptotic proteins, such as cytochrome *c*, are released that can activate downstream caspases.^43^ MOM permeabilization can be initiated by members of the Bcl-2 protein family, whose interactions either inhibit or promote cell death.^1,44^ Induction of apoptosis is associated with activation of pro-apoptotic family proteins, including Bax,^45,46^ whose activation is held in check by direct or indirect interaction with pro-survival Bcl-2 family members, including Bcl-xL.^47–50^

The conformation of cytoplasmic Bax is proposed to contain a hydrophobic groove into which the C-terminal transmembrane region is inserted, and a minor groove on the opposite side. The major hydrophobic groove is homologous to that on pro-survival proteins, such as Bcl-xL^51^ and the minor groove has a similar distribution of hydrophobicity and charge. The minor groove is masked by an unstructured loop, which is proposed to be a trigger site for Bax activation.^3^ Much evidence indicates that activation of Bax involves conformational changes^52,53^ initiated by displacement of the unstructured loop which then causes allosteric release of both the N-terminal end and the C-terminal helix from the hydrophobic grooves.^3–5,54^ Bax exists primarily as a monomer in the cytosol of healthy cells,^6^ but activated Bax translocates to the mitochondrial membrane^1^ where it is organized as assemblies of dimers.^55^ After its insertion into the MOM, Bax oligomerizes^8^ and Bax oligomers induce pore formation in the mitochondrial membrane.^11^ The ability of active Bax to form pores in the MOM, causing cytochrome *c* release, is presumed to be an essential initiating event in apoptotic pathways.^1^ The mechanism of pore formation is still unresolved, especially since conformational similarities between pro-apoptotic and pro-survival Bcl-2 proteins do not account for their functional differences.

The structure of Bcl-xL^56^ has a distinct similarity to the translocation domain of diphtheria toxin,^57^ a domain that can form pores in artificial lipid bilayers. However, even though it can be shown that Bcl-xL can form pores in lipid bilayers,^58^ it was recently shown that these are transient alterations that may have no consequences in mitochondrial membranes.^59^ In healthy cells, Bcl-xL is found in an equilibrium between the cytosol and intracellular membranes of mitochondria and other organelles.^60^ For the membrane-bound conformation of Bcl-xL, the unstructured loop touches the mitochondrial membrane surface, and there is speculation that this region could mediate membrane insertion of the protein.^61,62^ Phosphorylation of both Bcl-2 and Bcl-xL within this unstructured region induced conformation changes and changed their anti-apoptotic functions, although phosphomimetics (aspartic or glutamic acid for serine) and phosphorylation-defectives (alanine for serine) even at the same residue could have different results depending on the type of stress.^63–71^ None of the mutations converted these pro-survival proteins into constitutively active pro-apoptotic proteins, but rather had the effects of either weakening or enhancing interactions with pro-apoptotic proteins. The conformation and functional changes to Bcl-xL imparted by phosphorylation of the unstructured loop highlights the importance of this region to the ultimate function of the pro-survival protein.

The Cdk2 phosphorylation of Bcl-xL at serine 73 caused by cisplatin exposure resulted in changes to the Bcl-xL protein consistent with pro-apoptotic Bax activation, that is, that it changed the conformation of the protein, which can be seen as a slower migration of the phosphomimetic on PAGE (Figure 1d, lane 3). This phosphorylation converted the Bcl-xL into a pro-apoptotic protein, even without additional stress such as cisplatin exposure, and caused morphologic changes to the cells typical of apoptosis (Figure 2a), perinuclear mitochondrial clustering (Figure 2b), cytochrome *c* release from mitochondria (Figure 2c), caspase activation (Figure 2d, Supplementary Figure S3A) and cellular fragmentation (Supplementary Figure S3B). Conversely, a phosphorylation-defective mutant at serines 72, 73 and 74 protected from cisplatin (Figure 3, Supplementary Figure S3B).

The continuous repression of activated pro-apoptotic proteins by interaction with pro-survival proteins was found to be the most efficient mode of pro-apoptotic protein neutralization.^72^ In this model, apoptosis is initiated when all pro-survival proteins have been neutralized.^48,50^ We therefore examined the efficiency of S73D Bcl-xL for binding Bax in extracts both from control cells and from cells in which Bax was activated by tBID, an ‘activator’ Bcl-2 family protein (Figure 4A). The results indicated that it was unlikely that a decreased affinity between Bax and S73D Bcl-xL was responsible for cell death induction. The concentration of phosphomimetic Bcl-xL on the MOM (Figure 4B) is similar to the mitochondrial translocation of activated Bax. Also similar to active Bax, S73D Bcl-xL formed oligomers in the mitochondrial membrane (Figures 4C, panel a, lane 2), consistent with pore formation, that did not seem to need the participation of Bax (Figures 4C, panel b, lane 2). This demonstrated that the mechanism for the cell death induction by the phosphomimetic S73D Bcl-xL was its ability to oligomerize and form pores on the mitochondrial membrane, an action in which Bax did not participate.

A role for Bax activation in cisplatin cytotoxicity was demonstrated by the comparison of wild-type and Bax knock-out mice.^45^ The Bax-deficient mice were less susceptible to both functional damage (increased serum creatinine) and morphologic changes (increased tubular cell damage). Although we could not demonstrate higher molecular weight Bax oligomers associated with the mitochondrial membranes (Figures 4A and C, lanes 3, 4), we do not know to what extent the monomeric protein contributed to cell death.

We demonstrated an upstream role of Cdk2 in certain apoptotic pathways resulting in caspase activation and cell death. Many different cancers are dependent on Cdk activity for proliferation and Cdk inhibition is a target of chemotherapeutic drug development.^73^ The pathway described, in which Cdk activity results in cell death rather than survival, could potentially be exploited in cancer drug design.

## Materials and Methods

### Cell culture and treatments

Ad-293 cells (Stratagene, La Jolla, CA, USA) or HEK-293 cells (ATCC, Manassas, VA, USA) were grown at 37 °C in 5% CO_2_ in DMEM supplemented with 10% heat-inactivated fetal bovine serum. TKPTS cells were a gift from Dr. Bello-Reuss^74^ and were maintained as described previously.^31^ Mouse thick ascending limb cells (TAL) were a gift from Dr. GT Nagami (VA Medical Center, Los Angeles, CA, USA). Cisplatin was added to a final concentration of 25 *μ*M when the cells were ~75% confluent, and the cells were grown for an additional 20–24 h. Adenovirus was added 18 h before the addition of cisplatin. All adenoviruses were added where indicated to a final MOI of 100, which resulted in infection of over 95% of the cells. Where indicated, purvalanol was added to 9 *μ*M and zVAD was added to 5 *μ*M.

To visualize mitochondria, cells were grown on chamber slides before virus was added to 20 MOI. Two days after transduction, 100 nM MitoTracker Red CMXRos (Molecular Probes, Eugene, OR, USA) was added and the slides incubated 1 h at 37 °C. Cells were washed with PBS, fixed with 4% neutral buffered formaldehyde and covered with DAPI-containing mounting medium (Vector, Burlingame, CA, USA).

### Adenovirus

Recombinant viruses were generated by homologous recombination using the AdEasy^75^ vector system (supplied by Dr. B Vogelstein). As-Cdk2 adenovirus in which a glycine codon (GGG) was substituted for a phenylalanine codon (TTT) at amino acid position 80 in wild-type Cdk2 was constructed as described.^76^ Adenoviruses were amplified in HEK-293 cells, and purified by CsCl banding as described previously.^21^ CyclinA adenovirus was a gift from Dr. Gerald Denis (Boston Medical School, Boston, MA, USA).

### Site-directed mutagenesis

Site-directed mutagenesis was performed with the Q5 kit (New England Biolabs, Beverly, MA, USA) according to the manufacturer’s recommendations. Mutations were performed either in pBM272 Bcl-xL (mouse full-length Bcl-xL, Addgene) or in pET29b Bcl-xL (human residues 1–209 lacking the C-terminal hydrophobic region, containing a polyhistidine tag at the C-terminus).^56^ Primers for the S73D mutation were 5′-GTGGCTTTCACCGCG-3′ and 5′-TGGCCACAGCGACAGTTTGGATGCC-3′; for the S72, 73, 74A mutation were 5′-CGCTTTGGATGCCCGGGAGGTG-3′ and 5′-GCCGCGTGGCCAGTGGCTCCATTC-3′.

For construction of adenovirus expression vectors, full-length Bcl-xLs were excised from plasmids using *Bg*lII and *Eco*RV for insertion into pAdTrack-CMV.

### Western blot analysis

Proteins were extracted from TKPTS cells using RIPA lysis buffer containing 50 mM Tris-HCl, pH 7.5, 150 mM NaCl, 0.1% sodium dodecyl sulfate, 0.5% sodium deoxycholate, 1 mM EDTA with phosphatase inhibitor I and II and proteinase inhibitors (Sigma, Dallas, TX, USA). Extracts were sonicated and cell debris was removed by centrifugation for 10 min at 13 000 r.p.m. Protein concentration was determined using a Bio-Rad protein assay (Bio-Rad, Hercules, CA, USA). Western blot analyses were performed as described.^31^

Cytoplasm and mitochondrial fractions were prepared from cells scraped into hypotonic buffer containing 0.3 M sucrose, 10 mM Hepes, pH 7.4, 1 mM EGTA, with proteinase inhibitor cocktail (Sigma). The cells were disrupted using a Potter–Elvehjam teflon–glass homogenizer (Eberbach, Ann Arbor, MI, USA) and nuclei and unbroken cells were separated from the post-nuclear fraction by centrifugation at 1000×*g*, for 10 min. Mitochondria were pelleted by centrifugation at 7000×*g*, 15 min and resuspended in hypotonic buffer. Prior to electrophoresis, protein concentrations were determined as above and the samples were boiled in Laemmli buffer.^77^

The efficiency of Bax binding to wild-type or S73D Bcl-xL was measured using an assay in which Bax, either unactivated or activated by tBid-expression adenovirus (a gift from Dr. A Gross, Weizmann Institute of Science, Rehovot, Israel) was bound to anti-Bax antibody (Trevigen, Gaithersburg, MD, USA) on magnetic Protein-G-Dynabeads, and used to immunoprecipitate Bcl-xL. Bcl-xLs were GFP fusion proteins and were derived from His-tagged ΔC Bcl-xL. TKPTS cells were used as a source of Bax, and TAL cells that do not contain endogenous Bcl-xL were transfected with Bcl-xL expression plasmids, and used as a source of Bcl-xL. TKPTS and TAL cells were recovered, mixed together, resuspended in buffer with 10 mM Hepes, pH 7.4, 1 mM EDTA, and proteinase inhibitor cocktail and disrupted using a Potter–Elvehjam homogenizer. The post-nuclear fraction, isolated as above, was adjusted to 150 mM NaCl, 50 mM Tris, pH 7.4 and incubated at 4 °C for 18 h on a shaker with anti-Bax Dynabeads. Unbound proteins were removed by washing with Tris-saline and bound proteins eluted in Laemmli buffer.

### Blue-native gel electrophoresis

Three days after transduction of TKPTS cells with Bcl-xL expression adenovirus, proteins were isolated and separated into cytoplasmic and mitochondrial fractions as described. Mitochondrial proteins were solubilized by extraction with 1% *n*-dodecyl-*β*-*D*-maltoside. Cytoplasmic and mitochondrial proteins were separated by Blue Native PAGE using 4–16% Native PAGE Bis-Tris gels according to the protocol by the manufacturer (Life Technologies, Carlsbad, CA, USA).

### Antibodies

The antibodies used are as follows: caspase-3 (9661 and 9662, Cell Signaling, Beverly, MA, USA), Bcl-2 (ab7973, Abcam, Cambridge, MA, USA), Mcl-1 (ab53709, Abcam), Bcl-xL (AF800, R&D Systems, Minneapolis, MN, USA), cytochrome *c* (556433, BD Biosciences, San Diego, CA, USA), Bax (2772, Cell Signaling; 2280, Trevingen), L-aromatic amino acid decarboxylase (AV41425, Sigma), Thiophosphate ester (ab92570, Abcam), Cdk2 (ab7954, Abcam, polyclonal rabbit), cytochrome *c* Oxidase (459600, Invitrogen, Carlsbad, CA, USA) and Caspase-7 (9492, Cell Signaling)

### Assays for apoptosis

#### *Light microscopy*

Cells were photographed using an inverted microscope (Nikon Eclipse TE200, Melville, NY, USA) using Hoffman modulation contrast optics, ×10/0.25 NA objective, with a Nikon CoolPix 990 camera (Nikon) before harvesting.

#### *Cytochrome c release from mitochondria*

Cytoplasm and mitochondrial fractions were prepared from cells as above and western blots were used to detect cytochrome *c*. Caspase-7 was used as a cytoplasmic marker and cytochrome *c* oxidase was used as a mitochondrial marker.

#### *Caspase-3 activation*

Three days after adenoviral transduction, TKPTS cells were lysed in RIPA buffer as above and analyzed by western blot for pro-caspase-3 and activated caspase-3.

### Determination of Cdk2 substrates

#### *Identification of Bcl-xL as substrate*

Generation of as-Cdk2 adenovirus, transduction of cells with as-Cdk2 and cyclinA adenovirus, immunoprecipitation of active as-Cdk2/cyclinA and synthesis of radiolabeled analog *N*^6^-benzyl-[*γ*-^32^P]ATP was as described.^76^ Radiolabeled analog ATP was synthesized by a phosphate transfer reaction using *N*^6^-benzyl-ADP (a gift from Dr. DO Morgan, the University of California, San Francisco, CA, USA) and nucleotide diphosphate kinase (Sigma). Briefly, the as-Cdk2/cyclinA was prepared from 500 *μ*g post-nuclear supernatant protein isolated from as-Cdk2 and cyclinA adenovirus-infected TKPTS cells with and without cisplatin treatment (25 *μ*M, 24 h). Protein substrates were extracted from TKPTS cells and dialyzed twice against 1 l kinase buffer (20 mM HEPES, pH 7.5, 10 mM MgCl2 without DTT) containing 10 mM benzamidine as a protease inhibitor. After dialysis, DTT was adjusted to 1 mM. The proteins from 100 *μ*g cell lysate were kinased using immunoadsorbed as-Cdk2/cyclinA (100 ng) and 10 *μ*Ci *N*^6^-benzyl-[*γ*-^32^P]ATP. The reaction was incubated at 30 °C for 30 min. The proteins were dissociated with 1% SDS, 0.2 M DTT and precipitated with acetone. Protein pellets were redissolved in 8 M urea, 2% CHAPS, 1 M thiourea, 50 mM DTT and 0.2% Biolytes. The proteins were resolved in the first dimension by isoelectric focusing using the Bio-Rad Protean IEF Cell #165–4000 (Bio-Rad) and ReadyStrip IPG strips (Bio-Rad) with broad range pI resolution (pH range 4–7) followed by reducing 8–16% SDS-PAGE for the second dimension. All of the procedures were according to the manufacturer. Proteins that could be visualized by silver staining and overlaid a spot of radioactivity were identified by mass spectrometry.

#### *Identification of phosphorylation site in Bcl-xL*

Human Bcl-xL lacking the putative transmembrane region and containing a 6 amino acid C-terminal His tag (pET29b Bcl-xL)^56^ was obtained from Dr. T Chambers (the University of Arkansas for Medical Sciences) and isolated from BL21(DE3) bacteria. Analog ATP in the *in vitro* kinase reaction used *N*^*6*^-benzyladenosine-5ʹ-O-(3-thiotriphosphate) (Axxora, San Diego, CA, USA) as precursor. Phosphorylation was confirmed by western analysis on aliquots alkylated by treatment with *p*-nitrobenzyl mesylate using anti-thiophosphate ester antibody. Thiophosphorylation in the peptide fragments were identified by mass spectrometry as sites of specific Cdk2 phosphorylation.

#### *Mass spectrometry*

SDS-PAGE gel spots were excised and subjected to in-gel trypsin digestion as follows. Protein-containing gel slices were destained in 50% methanol (Fisher, St. Louis, MO, USA), 100 mM NH_4_HCO_3_ (Sigma-Aldrich, Dallas, TX, USA), followed by reduction in 10 mM Tris(2-carboxyethyl)phosphine (Pierce, Rockford, IL, USA) and alkylation in 55 mM iodoacetamide (Sigma-Aldrich). Gel slices were then dehydrated in acetonitrile, followed by addition of 100 ng porcine trypsin (Promega, Madison, WI, USA) in 100 mM NH_4_HCO_3_ (Sigma-Aldrich) and incubated at 37 °C for 12–16 h. The trypsin solution was extracted and the gel slices were dehydrated in a SpeedVac (Holbrook, NY, USA). Chymotrypsin in 100 mM Tris-HCl, pH 7.8 containing 10 mM CaCl_2_ was added to gels. Gels were incubated at 37 °C for 4–6 h. Following the second enzyme digestion, peptides were extracted with 5% formic acid in 50% acetonitrile and dried in a SpeedVac. The peptide products were reconstituted with 0.1% formic acid, 2% acetonitrile, followed by MS/MS analysis using an LTQ XL mass spectrometer (Thermo, San Diego, CA, USA). Proteins and modifications are identified from MS/MS spectra by database searching Mascot (a trademark of Matrix Science, Inc., Boston, MA, USA) and SEQUEST using search engines. The analysis was performed by L Li (Institute of Molecular Medicine, the University of Texas Health Center, Houston, TX, USA).

### Statistical analysis

Statistical analysis was performed with ANOVA and a *t*-test. Results were expressed as means±S.E.M. *P*<0.01 was considered significant.

## Abbreviations

as-Cdk2: analog-sensitive Cyclin-dependent kinase 2
Cdk2: Cyclin-dependent kinase 2
MOM: mitochondrial outer membrane
TAL: mouse kidney thick ascending limb cells
TKPTS: mouse kidney proximal tubule epithelial cells.
