# Supplementary material for: Cdk2 phosphorylation of Bcl-xL after stress converts it to a pro-apoptotic protein mimicking Bax/Bak
Source: Cell Death Discov. 2016 Jan 18;2:15066–. doi: 10.1038/cddiscovery.2015.66 (PMC4877050; doi:10.1038/cddiscovery.2015.66)
Supplement: Supplementary Figures [file cddiscovery201566-s1.pdf]

Figure S1

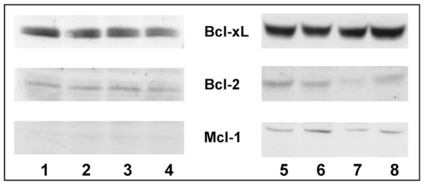

Figure S2A

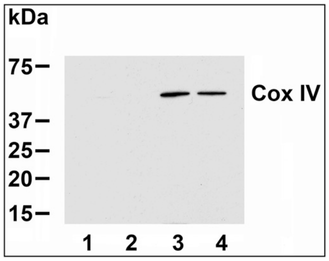

Figure S2B

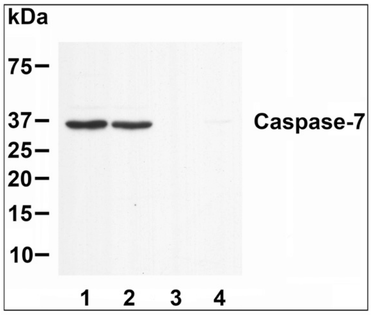

Figure S3A

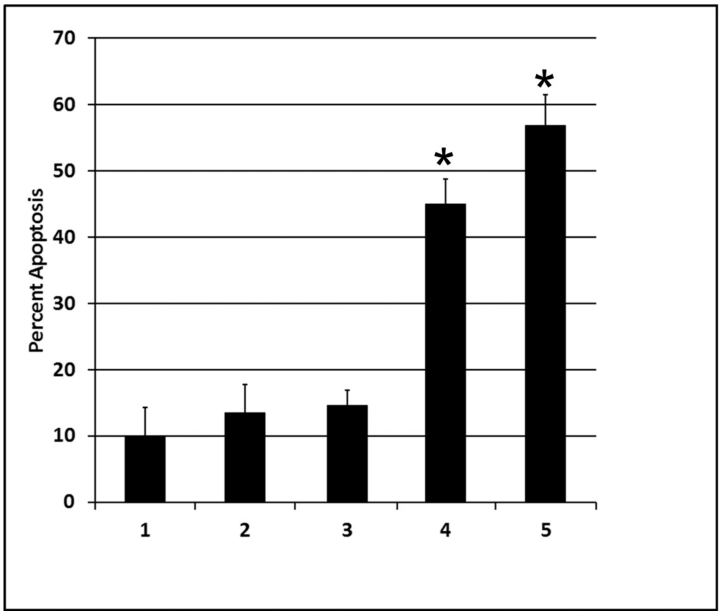

Figure S3B

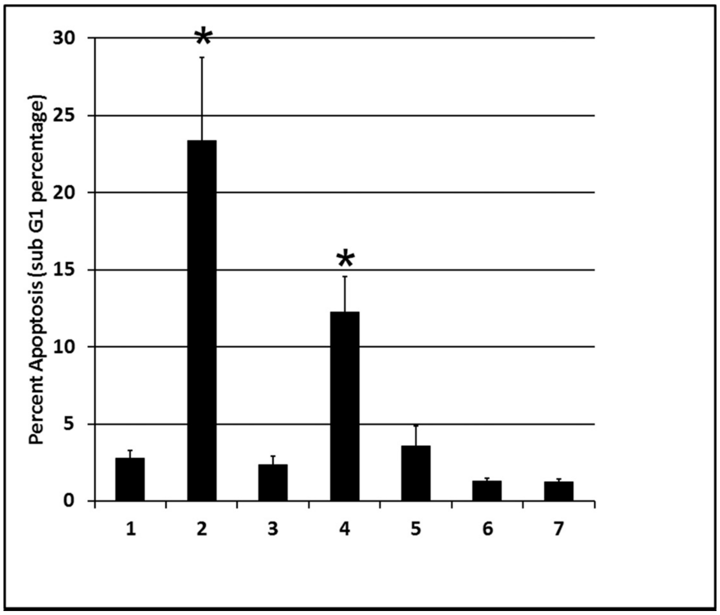

SUPPLEMENTAL FIGURE LEGENDS

Figure S1. Relative amounts of pro-survival Bcl-2 family proteins. Proteins isolated from TKPTS cells (lanes 1-4) and mouse kidney extracts (lanes 5-8). Protein extracts (100 μg/lane) were electrophoresed on 10% PAGE and Bcl-2 family proteins detected by western blotting and ECL. Cisplatin was administered to cells (25 μM) for 0 hrs (lane 1), 3 hrs (lane 2), 6 hrs (lane 3), or 9 hrs (lane 4) or injected into mice (IP, 20 mg/kg) for 0 hrs (lane 5), 1 day (lane 6), 2 days (lane 7), or 3 days (lane 8) before kidneys were collected.

Figure S2A. Immunoblot for relative purity of mitochondrial fraction. Cytochrome C oxidase, complex IV subunit I immunoblot used as a marker for mitochondrial fraction for subcellular fractions in Figures 2C and 4B. Cells fractionated into cytoplasmic (lanes 1, 2) and mitochondrial (lanes 3, 4) fractions 3 days after transduction with adenovirus expressing wild-type (lanes 1, 3) and S73D Bcl-xL (lanes 2, 4). For the cytoplasmic fraction, 100 μg protein was loaded, and for the mitochondrial fraction, 10 μg protein was loaded.

Figure S2B. Immunoblot for relative purity of cytoplasmic fraction. Caspase-7 immunoblot used as a marker for cytoplasmic fraction for subcellular fractions in Figures 2C and 4B. Cells fractionated into cytoplasmic (lanes 1, 2) and mitochondrial (lanes 3, 4) fractions 3 days after transduction with adenovirus expressing wild-type (lanes 1, 3) and S73D Bcl-xL (lanes 2, 4). For the cytoplasmic fraction, 100 μg protein was loaded, and for the mitochondrial fraction, 10 μg protein was loaded.

Figure S3A. Caspase activation indicated by Red-VAD binding. Control cells (lane 1; 10.11±4.28% Red-VAD labeled cells) were not infected but were cultured for the same time as adenoviral infected cells. Cells were infected with either wild-type Bcl-xL (lane 2; 13.52±4.19%),

S72,73,74A Bcl-xL (lane 3; 14.68±2.28%), or S73D Bcl-xL (lane 4; 45.07±3.69%). One cell culture was also treated with 25 μM cisplatin (lane 5; 56.92±4.55%) and harvested 24 hours after exposure. Asterisk, $p<0.01$ compared with untreated control. Error bars indicate S.E.M.

Figure S3B. Apoptosis induction by S73D Bcl-xL indicated by cell cycle analysis by FACS. Cells were infected with Bcl-xL expression adenoviruses and cultured for 3 days. After fixing and staining with propidium iodide, the cells were analyzed by FACS and cells in sub G_1G_0 were selected as apoptotic. Cells were either untreated (lane 1) or exposed to cisplatin (lane 2), which resulted in 2.80±0.48% or 23.37±5.37% cells in apoptosis, respectively. Cells infected with Bcl-xL expression adenoviruses (lanes 3-7), in which expression was wild-type (lane 3, 2.35±0.60% in apoptosis), S73D (lane 4, 12.25±2.31%), or S72,73,74A (lanes 6, 1.28±0.22%). In addition, some cells expressing S73D were supplemented with pan-caspase inhibitor zVAD-fmk (lane 5, 3.56±1.29%), and some cells expressing S72,73,74A was exposed to cisplatin (lane 7, 1.24±0.16%). Asterisk, $p<0.01$ compared with untreated control. Error bars indicate S.E.M.

SUPPLEMENTAL EXPERIMENTAL PROCEDURES

Proteins were extracted from kidneys by homogenization in RIPA lysis buffer as described. The extract was sonicated (3 x 30 sec), followed by centrifugation at 13,000 x G, 10 min. Protein concentration in the supernatant was determined and electrophoresis was performed at described. Western blot analyses were performed as described (Price et al., 2004). Protein was electrophoresed through 12% SDS-polyacrylamide gel and transferred to polyvinylidene difluoride membranes. After blocking with 5% nonfat dry milk in TBST, the membrane was incubated at 4°C overnight with primary antibody. After washing, horseradish peroxidase-congugated secondary antibody was applied. Proteins that bound to the secondary antibody were visualized using enhanced chemiluminescence (Amersham Biosciences).

Red-VAD analysis. Cells were transduced with adenoviruses and harvested after 3 days using the QIA92 caspase detection kit (EMD Millipore) according to the manufacturer's protocol. Briefly, the pan-caspase inhibitor VAD-fmk conjugated to sulfo-rhodamine (Red-VAD) was added and the cells returned to the incubator for an additional hour. After harvesting and washing to remove unbound Red-VAD, cell were resuspended in 300 μl labeling buffer and analyzed by flow cytometry. For cells infected with adenovirus, coexpression of GFP was used as an indicator of infected cells. Analyses were performed on at least 4 independent samples. This analysis is not specific for any one caspase, but rather Red-VAD binds irreversibly to most activated caspases.

FACS analysis. The method used for cell cycle analysis by fluorescence-activated cell sorter (FACS) analysis was described previously (Price, et al., 2004). For each culture condition, at least 10,000 cells were analyzed and the analyses were performed on at least four separate cultures. The percentage of cells in sub-G_1/G_0 (apoptotic fraction; Darzynkiewicz and Gong, 1994) was determined using WinMDI 2.8.
